# Nanofibrillar cellulose-alginate hydrogel coated surgical sutures as cell-carrier systems

**DOI:** 10.1371/journal.pone.0183487

**Published:** 2017-08-22

**Authors:** Patrick Laurén, Petter Somersalo, Irina Pitkänen, Yan-Ru Lou, Arto Urtti, Jouni Partanen, Jukka Seppälä, Mari Madetoja, Timo Laaksonen, Antti Mäkitie, Marjo Yliperttula

**Affiliations:** 1 Division of Pharmaceutical Biosciences, Centre for Drug Research, Faculty of Pharmacy, University of Helsinki, Helsinki, Finland; 2 Department of Engineering Design and Production, School of Engineering, Aalto University, Espoo, Finland; 3 School of Pharmacy, University of Eastern Finland, Kuopio, Finland; 4 Made Consulting, Turku, Finland; 5 Department of Chemistry and Bioengineering, Tampere University of Technology, Tampere, Finland; 6 Department of Otorhinolaryngology—Head and Neck Surgery, University of Helsinki and Helsinki University Hospital, Helsinki, Finland; University of South Carolina, UNITED STATES

## Abstract

Hydrogel nanomaterials, especially those that are of non-human and non-animal origins, have great potential in biomedical and pharmaceutical sciences due to their versatility and inherent soft-tissue like properties. With the ability to simulate native tissue function, hydrogels are potentially well suited for cellular therapy applications. In this study, we have fabricated nanofibrillar cellulose-alginate (NFCA) suture coatings as biomedical devices to help overcome some of the limitations related to cellular therapy, such as low cell survivability and distribution out of target tissue. The addition of sodium alginate 8% (w/v) increased the NFCA hydrogel viscosity, storage and loss moduli by slightly under one order of magnitude, thus contributing significantly to coating strength. Confocal microscopy showed nearly 100% cell viability throughout the 2-week incubation period within and on the surface of the coating. Additionally, typical morphologies in the dual cell culture of spheroid forming HepG2 and monolayer type SK-HEP-1 were observed. Twelve out of 14 NFCA coated surgical sutures remained intact during the suturing operation with various mice and rat tissue; however, partial peeling off was observed in 2 of the coated sutures. We conclude that NFCA suture coatings could perform as cell-carrier systems for cellular based therapy and post-surgical treatment.

## Introduction

Classic biomaterials and biomedical devices are widely used and well established in combination with surgery and post-surgical treatment today [1]. However, recent developments have introduced new treatment strategies and possibilities, such as controlled and localized drug delivering sutures [2], and stem cell transplanting sutures [3,4]. These biomedical devices have great potential in improving patient treatment and recovery. However, it has been reported that there are some risks related to administering cells in current fashion, e.g. injections, or seeding the suture, can cause unwanted cell aggregation, in addition to cells migrating and distributing to undesired tissues and low cell survivability due to a lack of protection against the mechanical stress of the suturing itself [4,5]. Additionally, while the seeding of cells within the surgical thread could be improved by increasing the amount of microthreads in the fold [3], the total number of cells could still be limited by the surface area of the thread. Some of these limitations could be circumvented with the entrapment of cells within a protective layer, such as a hydrogel coating.

Hydrogel nanomaterials and nanocomposites have recently gained increasing interest in modern medicine, including biomedical and pharmaceutical applications as well as tissue engineering [6]. Nanofibrillar cellulose (NFC) is one of these materials [7,8], with versatility and inherent properties that have led to studies that utilize NFC hydrogels as a biomedical material, such as a scaffold that promotes three-dimensional cell culture or a drug-releasing matrix [9–14]. NFC fibers have inherent similarity to collagen fibers [9,15], great modification capabilities [16,17], high water content, pseudoplastic and thixotropic properties [18], and are considered as a biocompatible and non-toxic biomaterial [19–20]. Similarly to NFC, alginate is a widely studied biopolymer, known to be biocompatible, non-toxic and frequently used in tissue engineering [21–23]. Alginate is a linear polysaccharide found in species of brown algae, and forms gels in the presence of divalent ions [24], such as calcium and barium, where the positively charged ions can bind with the negatively charged glucuronic acid blocks. Alginate has been the most recognized material for cell encapsulation [25,26], while providing immune protection and allowing good metabolic functionality in a 3D culturing environment [27–29]. In addition, NFC hydrogel and alginate composition has been used in bioprinting [30], where it was reported that the addition of NFC improved the hydrogel structural fidelity, preventing the collapse of printed shapes. The shear thinning characteristics of NFC and alginate [9,31], are particularly desirable features, as they enable hydrogel injectability, and thus, the fabrication of nanocomposite NFCA threads and suture coatings.

In this study, we have employed a method of producing nanofibrillar cellulose-alginate hydrogels (NFCA) in the form of threads and suture coatings for potential applications in cellular therapy. The fabricated NFCA suture coatings are an improvement to our groups previous glutaraldehyde cross-linked, slightly more brittle, threads [32], and might offer a solution to some of the limitations and risks related to cell delivery by acting as a cell-carrier system without the need for additional injections or separate cell transplantation procedures. Sutures could be placed at the target site while retaining cells within the coating matrix reducing cell distribution to unwanted tissues and functioning as a protective layer against the mechanical stress induced by the suturing process to improve cell survivability.

We have investigated the rheological properties of the NFCA hydrogels in addition to cell viability and suturing performance of the NFCA coated sutures on various tissues of rat and mouse. Cell studies were performed with HepG2 (cluster type) and SK-HEP-1 (monolayer type) cell lines as a co-culture model for carrying cells to the target site within the suture coating and on the surface respectively. Potential applications for such NFCA hydrogels are: cell-carrier systems including oral mucosa repair [33,34], ulcer treatment [35,36], and for example Crohn’s disease, which has been recently investigated in treatment of fistulas with adipose-derived stem cells [37–39], where cell transplantations were done with separate injections at the sutured internal openings of the fistulas. Additionally, cell co-culture systems enable different cell types to be utilized in a single treatment system [40], or a model that can be utilized in studies evaluating treatment efficiencies [41].

## Materials & methods

### NFCA thread preparation

NFC hydrogel and alginate mixtures were prepared to produce NFCA threads. Sodium alginate powder (Sigma-Aldrich, W201502, Finland) was added and mixed briefly with the stock NFC hydrogel (1.47% NFC, GrowDex®, UPM-Kymmene Corporation, Finland). After mixing, the mixture was left to stabilize for 24 hours. The final composition of NFCA prepared for cell studies and suturing performance tests contained 8% (w/v) sodium alginate and 1.35% (w/v) NFC hydrogel. The NFCA threads were prepared by dispensing the mixture with a syringe and a 22G needle into calcium chloride (CaCl_2_, anhydrous, Riedel-de-Haen, Germany) and barium chloride (BaCl_2_ * 2H_2_O, Sigma-Aldrich, Finland) crosslinking solutions (68 mM and 20 mM respectively). Alginate crosslinking method used in this study was implemented from an alginate-based cell microencapsulation study performed by one of our group previously [42].

### NFCA rheology

Rheological measurements were done with Haake™ Viscotester™ iQ Rheometer (ThermoFischer, Germany) using 2° cone-plate geometry. Plate diameter was 35 mm and the gap was set to 0.1 mm. Experiments were done at 25°C with peltier temperature controlled system. Oscillatory stress amplitude sweeps were performed to determine sample linear visco-elastic regions (1*10^−4^–500 Pa at the frequency of 1 Hz). The effect of frequency on storage and loss moduli was measured with a frequency sweep (0.1–20 Hz at constant amplitudes of 8 and 12 Pa for native NFC and NFCA respectively), and the viscosity was measured with the controlled rate mode (0.1–1000 1/s). Sample materials for rheological experiments included 1.35% (w/v) NFC hydrogel mixtures with 3 different amounts (7, 8 or 9% (w/v)) of sodium alginate. All measurements were replicated 3 times and averages shown with their respective standard deviations.

### Cell lines

Human hepatocellular carcinoma HepG2 (ATCC® HB-8065™, USA) and human adenocarcinoma-derived endothelial SK-HEP-1 cells (ATCC® HTB-52™, USA) were cultured in high glucose Dulbecco's modified Eagle's medium (DMEM, Gibco™, Scotland) supplemented with 10% fetal bovine serum, 100 U/ml penicillin, 100 μg/ml streptomycin, 2 mM L-glutamine and 100 mM sodium pyruvate. Cell cultures were maintained in 37°C and 5% CO_2_ until 70–80% confluence before detachment and preparation of cell suspensions. HepG2 cells were used as a model type for 3D cell organoids, and SK-HEP-1 cells for epithelial 2D monolayer culture.

### NFCA thread cell seeding and culturing

HepG2 cells were detached, centrifuged and suspended within the NFCA mixture. The NFCA-HepG2 mixture contained 1043 cells/μl (150 μl) and the final concentrations were 8% and 1.35% of sodium alginate and NFC respectively. NFCA crosslinking was performed by extruding the NFCA-HepG2 mixture through a 22G-size needle into a 68 mM calcium chloride solution and incubated for 3 minutes. The produced thread-like structures were transferred into a 20 mM barium chloride solution for additional 5 minutes. Calcium chloride and barium chloride crosslinking stabilized the hydrogel-thread structure to withstand handling in its wet state. Afterwards the threads (n = 5) were transferred into non-treated 6-well culture plates with 2 ml of full cell-culture medium and incubation was carried out at 37°C and in 5% CO_2._ Cell culture medium exchange was performed every 48 hours.

### NFCA thread cell surface and co-cultures

To enable co-culturing, the prepared NFCA-HepG2 threads were treated with Type-I collagen to enhance surface adhesion (Rat Collagen I (LV) 3 mg/ml, Cultrex®, Trevigen, USA). The collagen working solution was prepared according to the manufacturer’s guidelines with DMEM replacing water to dilute the stock solution. The concentration of Type-I collagen in the working solution was 1 mg/ml. The collagen working solution (300–500 μl) was pipetted on the NFCA or NFCA-HepG2 thread structures to cover them fully. The threads were soaked briefly in the collagen working solution and placed into a cell culture incubator for 30 minutes to allow collagen gel formation. After incubation, the threads were transferred into non-treated low attachment culture plates for HepG2 or SK-HEP-1 cell seeding. Cells were detached and suspended in 2 ml of full cell culture medium (1.2 * 10^6^ cells per ml). The cell suspension was introduced on top of the NFCA or NFCA-HepG2 threads and incubated for 2 hours. During the 2-hour incubation period the well plates were subsequently shaken every 30 minutes to facilitate cell attachment on the thread surface. After cell seeding, the well plates were placed into 37°C in 5% CO_2_ and incubated from 48 hours and up to 2 weeks until confocal imaging (n = 6 per each cell line). Cell culture medium was replaced every 48 hours.

### Suture coating with NFCA-cell mixtures

Surgical sutures coated with NFCA were prepared prior to soft tissue suturing attempts. Biodegradable synthetic polyester surgical sutures (Velosorb™ Fast, 3–0, Covidien, USA) were coated with NFCA-HepG2 mixtures using a syringe. The surgical suture was inserted through the syringe barrel and needle orifice. The syringe was filled with NFCA-HepG2 mixture and fed gently through the needle. The surgical suture was pulled at a slow rate to ensure the formation of an even layer of the NFCA-cell mixture on the surface of the surgical suture (1.5 * 10^4^ cells/cm/suture). The readily coated sutures (n = 6) were transferred in crosslinking solutions as described above to stabilize the NFCA coating. Directly after the crosslinking, each suture was sewn through commercially bought pig liver sections three times and analyzed with confocal microscopy. Suture weight was measured before and after the coating (AS 3Y Analytical Balance, Radwag, Poland; n = 5).

### Confocal microscopy

NFCA threads and related cell studies were examined and analyzed with confocal microscopy (Leica TCS SP5II HCS A, Leica Microsystems, Germany). Images were taken with HC PL APO 10x/0.4 objective using Red (HeNe 633 nm/12mW) and Lime (DPSS 561 nm/20mW) lasers with PTM detectors. Live/Dead imaging was performed with fluorescein diacetate (FDA) and propidium iodide (PI) cellular stains (Molecular Probes®, USA) for live and dead cells, respectively. Cell co-culture imaging was carried out with cellular dyes CellTracker™ Green CMFDA and Red CMTPX (Molecular Probes®, USA) for SK-HEP-1 and HepG2 cells, respectively. Suture and NFCA coating diameters (5 x 20 cm sutures) were measured using Leica LAS AF lite imaging suite (Leica Microsystems, Germany). 2 random positions were selected from each coated suture and diameter measured (n = 10).

### Suture performance *ex vivo* small-animal study

The small-animal *ex vivo* suture performance study was performed as a contract service by Made Consulting (Toxicology Partners, preclinical services, Finland), and approved by the Finnish National Animal Experiment Board and performed in accordance with the Animal Welfare Act (247/1996) according to the laws of Finland. Two BALB/c mice and a Wistar rat were sacrificed using a standard carbon dioxide chamber euthanasia method, and used to evaluate the performance of the surgical sutures coated with NFCA. Sutures were coated with NFCA and cross-linked as described above. Target soft tissue was sutured and instrument type knots were performed to complete the sutures (n = 14). Mouse liver, spleen, intestine, muscle and skin in addition to rat intestine, testis and skin were selected for the performance testing.

## Results

### NFCA hydrogel rheological properties

**Fig 1:**
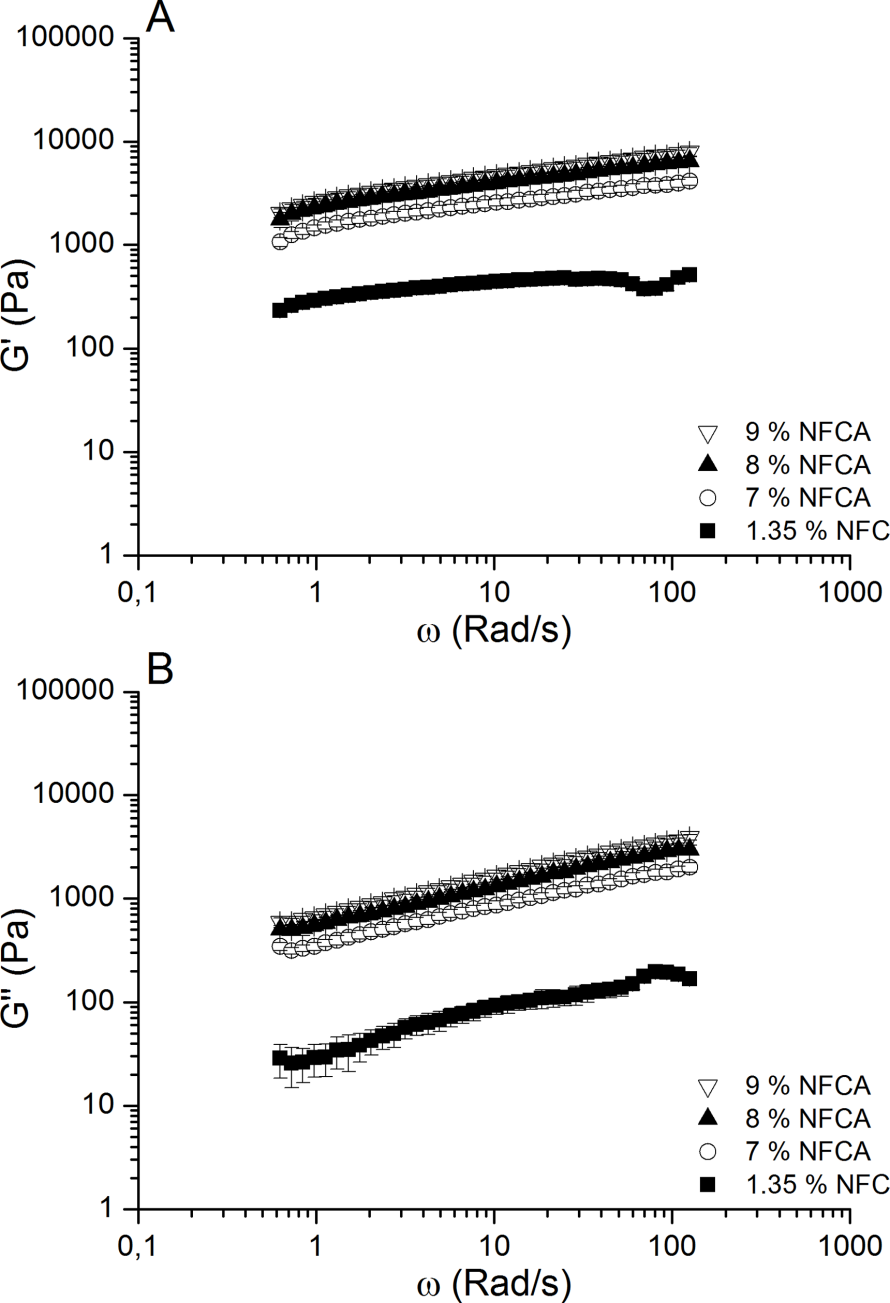
Frequency sweeps of NFCA samples. A) Storage modulus (G’) and B) loss modulus (G”) values showed increased strength in the NFCA network with the addition of alginate. NFCA hydrogels were relatively independent of angular frequency, which indicates a more stable gel structure as opposed to native NFC, where the values shift towards higher frequencies.

The prepared NFCA threads were able to withstand handling during the cell studies and surgical suture coating experiments with soft tissue sutures. Rheological measurements were conducted to observe how sodium alginate content affected the properties of the hydrogel. Viscosity and shear thinning are important factors for hydrogel injectability, and changes in storage and loss moduli could affect cell behavior, such as morphology. Frequency sweep measurements of storage and loss moduli were plotted as a function of angular frequency (Fig 1). The storage moduli were greater than loss moduli for all samples, and the loss tangent ranged 0.13–0.36 for NFC and approximately 0.29–0.49 for other samples. This suggests an elastic network structure of hydrogels. NFCA samples showed roughly 6-8-fold higher storage and loss moduli values, strengthening the hydrogel mixture network when compared to native NFC. This was due to the addition of alginate, which additionally enabled crosslinking with cationic salt treatment. As far as we know, this is the first time a non-breakable and flexible NFCA hydrogel coated thread has been prepared for surgical purposes.

**Fig 2:**
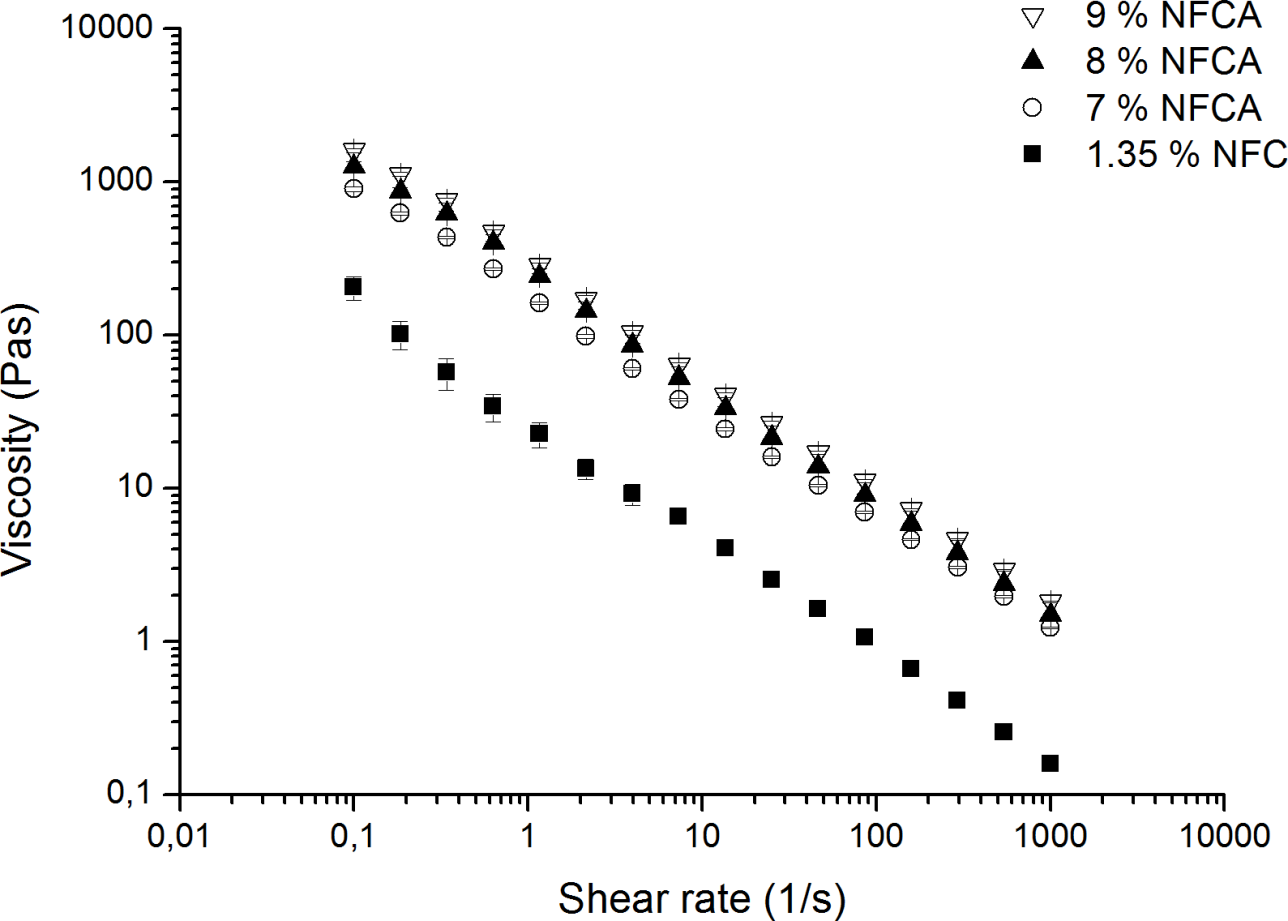
Viscosity measurements of NFC and NFCA samples. Addition of alginate increases the viscosity of the hydrogel composition. A minor increase can be observed with the incremental addition of alginate content within the mixture.

The viscosity increased significantly, when sodium alginate was included (Fig 2). Shear thinning was observed on all hydrogel samples, and a similar decline in viscosity values was seen when shear rate was gradually increased. This indicates similar shear thinning properties of the native NFC and NFCA hydrogels. The amount of alginate had some impact in the viscosity values when comparing changes within 1% increments, however 9% alginate content showed nearly double the value (44%) when observed against the 7% NFCA mixture.

### NFCA cell studies

Two different cell lines were selected to model 3D spheroid formation and monolayer growth within the wire and on the surface respectively. These cell lines (HepG2 and SK-HEP-1) represented different cell types that could be utilized in cell delivery via the surgical thread.

**Fig 3:**
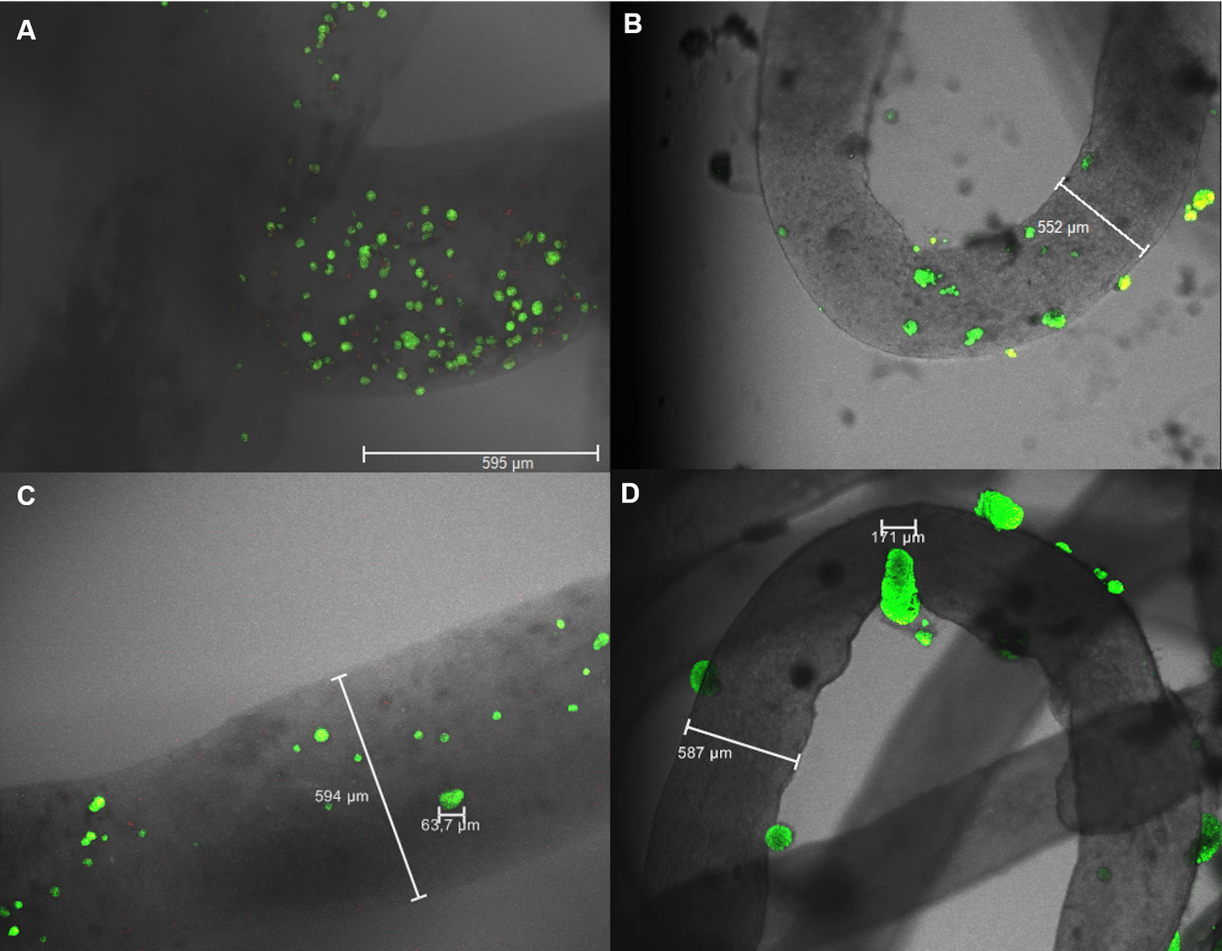
Live/Dead confocal imaging of NFCA-HepG2 threads stained with FDA (alive/green) and PI (dead/red). A) HepG2 cells were mixed within the NFCA before crosslinking; after 1-week of incubation and C) after 2 weeks of incubation. B) HepG2 cells were seeded on top of type-I collagen treated NFCA threads; after 1-week of incubation and D) after 2 weeks of incubation. An increase in the number of cells after the 2-week incubation period was not observed when the cells were mixed within the NFCA material. However, HepG2 clusters were growing on the surface cultures. Only a few dead cells were observed during the study period.

NFCA threads were seeded with hepatocellular carcinoma HepG2 cells and incubated for up to 2 weeks. Live/dead confocal microscopy images showed near 100% cell viability within and on the surface of the NFCA threads, and only a few dead cells (< 1%) were observed within the thread in both 1-week and 2-week incubation periods (Fig 3).

No dead cells were found on surface seeded NFCA threads; however, some variation in cell growth properties was observed depending on the seeding site. HepG2 cells on the surface showed typical cluster like growth and hepatic morphology. Cells seeded within the threads were observed to grow individually or only in small clusters. Dead cell staining was verified by killing the culture with a 70% ethanol treatment before confocal microscopy showing a 100% rate for dead cells S1 Fig.

**Fig 4:**
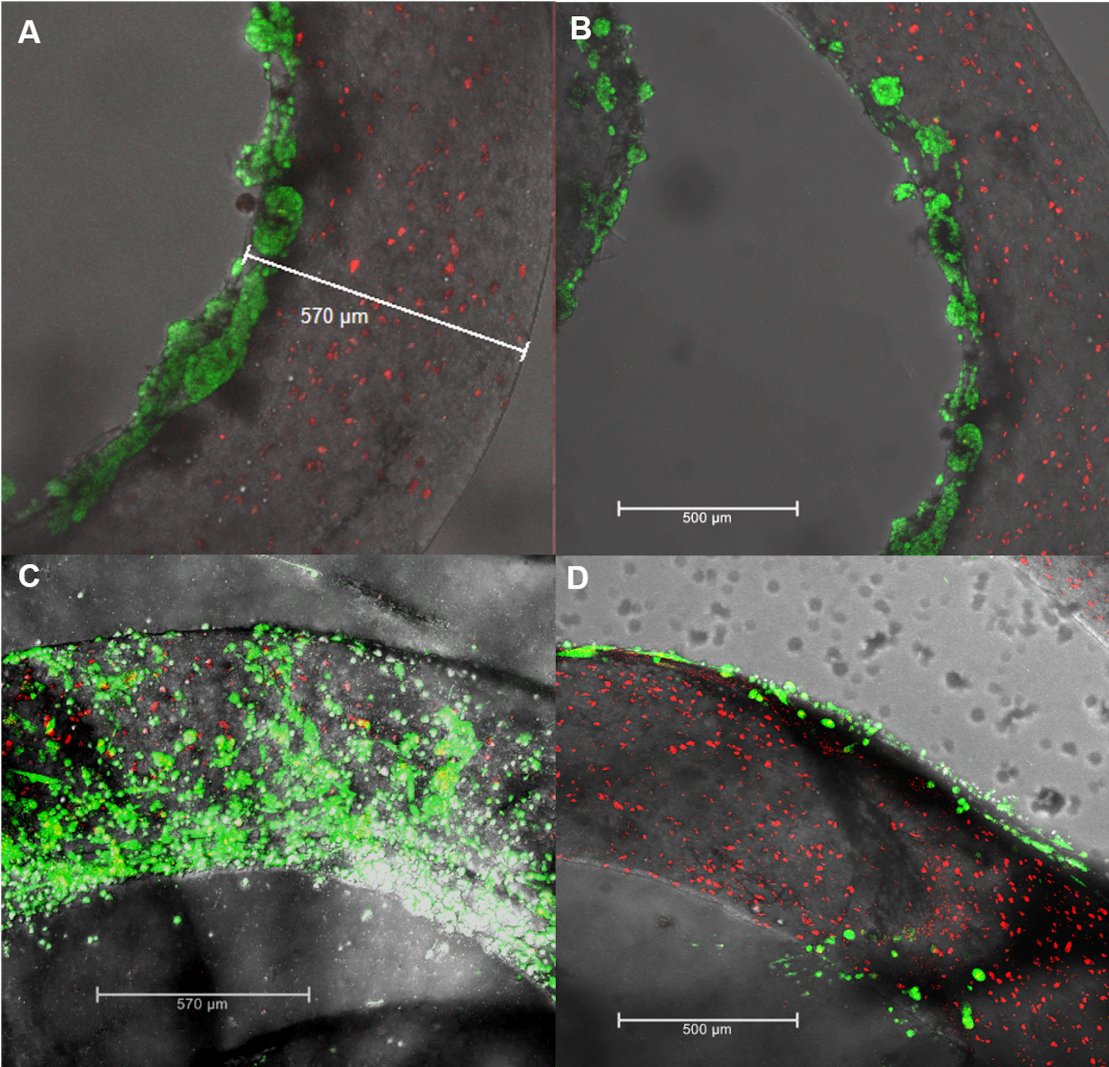
Confocal imaging of HepG2 and SK-HEP-1 cells stained with CTred and CTgreen, both markers for live cells. Type-I collagen treated NFCA threads with HepG2 cells mixed within the material (red staining/HepG2 cells within the thread). A-B) HepG2 cells seeded on the surface of the NFCA threads and incubated for 72h (green). C-D) SK-HEP-1 cells seeded on the surface of the NFCA threads and incubated for 48h (green). HepG2 cells within the threads grew individually or in very small clusters; however, surface growth showed typical cluster and epithelial morphology of HepG2 and SK-HEP-1 respectively.

HepG2 cells were seeded within and on the surface of the threads and incubated for 72 hours (Fig 4A and 4B). Similarly, to the live/dead viability experiment, cells within the thread grew individually or in small clusters containing only a few cells. Cells on the surface showed strong growth of hepatic-type clusters, typical for the HepG2 cell line.

Another human adenocarcinoma-derived endothelial cell line (SK-HEP-1) was added to investigate the NFCA thread co-culture surface growth properties. SK-HEP-1 cells were seeded on the surface of the NFCA threads in addition to the HepG2 cells within the threads and incubated for 48 hours. As opposed to the cluster like HepG2 morphology, SK-HEP-1 cell growth showed epithelial-like morphology which is typical for the endothelial SK-HEP-1 cell line (Fig 4C and 4D). However, it should be noted that without collagen coating neither SK-HEP-1 nor HepG2 adhered properly on the surface of bare NFCA threads. Collagen coating was not required for cells that were entrapped within NFCA.

### NFCA coated surgical sutures

**Fig 5:**
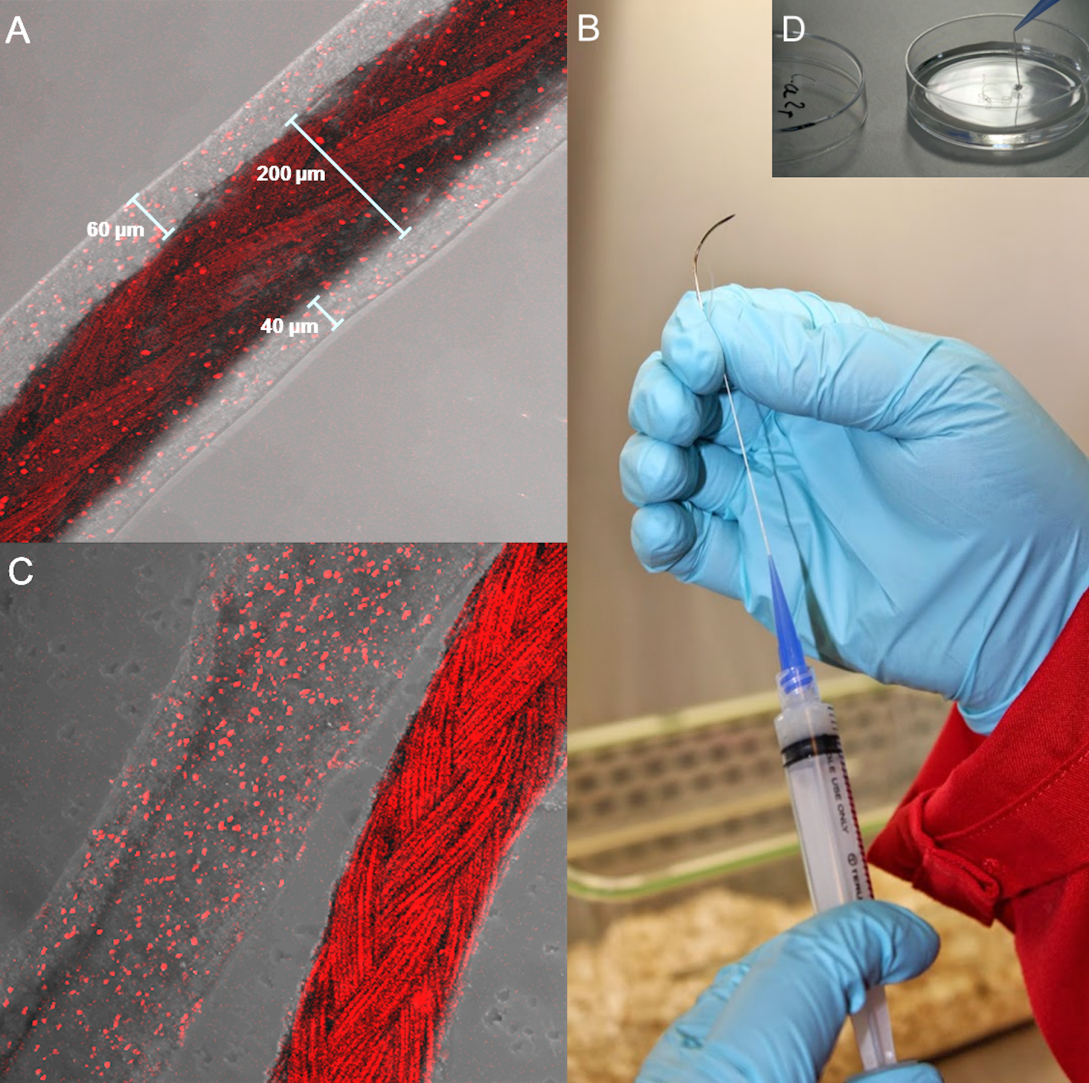
Confocal imaging and preparation of NFCA-HepG2 coated surgical sutures. Live staining of CTred was performed after 72 h incubation. A) Sutures coated with NFCA-HepG2 and sewn three times through a pig liver segment indicating live HepG2 cells within the coating matrix. B, D) Preparation method of NFCA coated sutures. C) NFCA-HepG2 coating could be peeled off from the surgical suture as intact segments without damaging its tube-like structure showing live HepG2 cells remaining within the coating.

HepG2 cells were seeded within the NFCA mixture, extruded through a syringe needle as a layer on the surface of standard absorbable surgical sutures (Fig 5B) and incubated for 72 hours. HepG2 growth was shown, as previously observed, as small clusters or individual cells (Fig 5A). NFCA-HepG2 coating was shown as an even layer on top of the suture; however, slight inconsistencies were found when examining the thickness of the coating around the suture. According to the United States Pharmacopeia (USP), the diameter of a standard 3–0 absorbable suture is 200 μm. Surrounding the suture, the average NFCA coated suture thickness was measured at 297±25 μm in diameter (n = 10). Average weight of the threads (n = 5) before and after coating were 0.86±0.04 mg/cm and 1.96±0.27 mg/cm respectively, a total increase in weight of 1.1 mg/cm for the coated suture. The extruded pure NFCA hydrogel required a force from the plunger, therefore, the product of a thread like hydrogel was thicker (a total diameter of 550–600 μm; n = 5) than the coated sutures. The NFCA coating fabrication did not require any force from the plunger as the suture was pulled through the nozzle; therefore, producing thinner coatings than the NFCA hydrogel threads themselves without the suture.

The NFCA-HepG2 coated sutures were used to simulate sewing three times through small segments of pig liver. The coating remained intact on top of the suture thread after sewing (Fig 5A). Additionally, HepG2 cells were confirmed alive within the coating with confocal microscopy. The NFCA-HepG2 coating could be removed or peeled off as tube-like long segments with the entire NFCA structure intact, indicating that the cells were entrapped within the coating matrix (Fig 5C).

### NFCA thread sutures on *ex vivo* small-animal tissue

**Fig 6:**
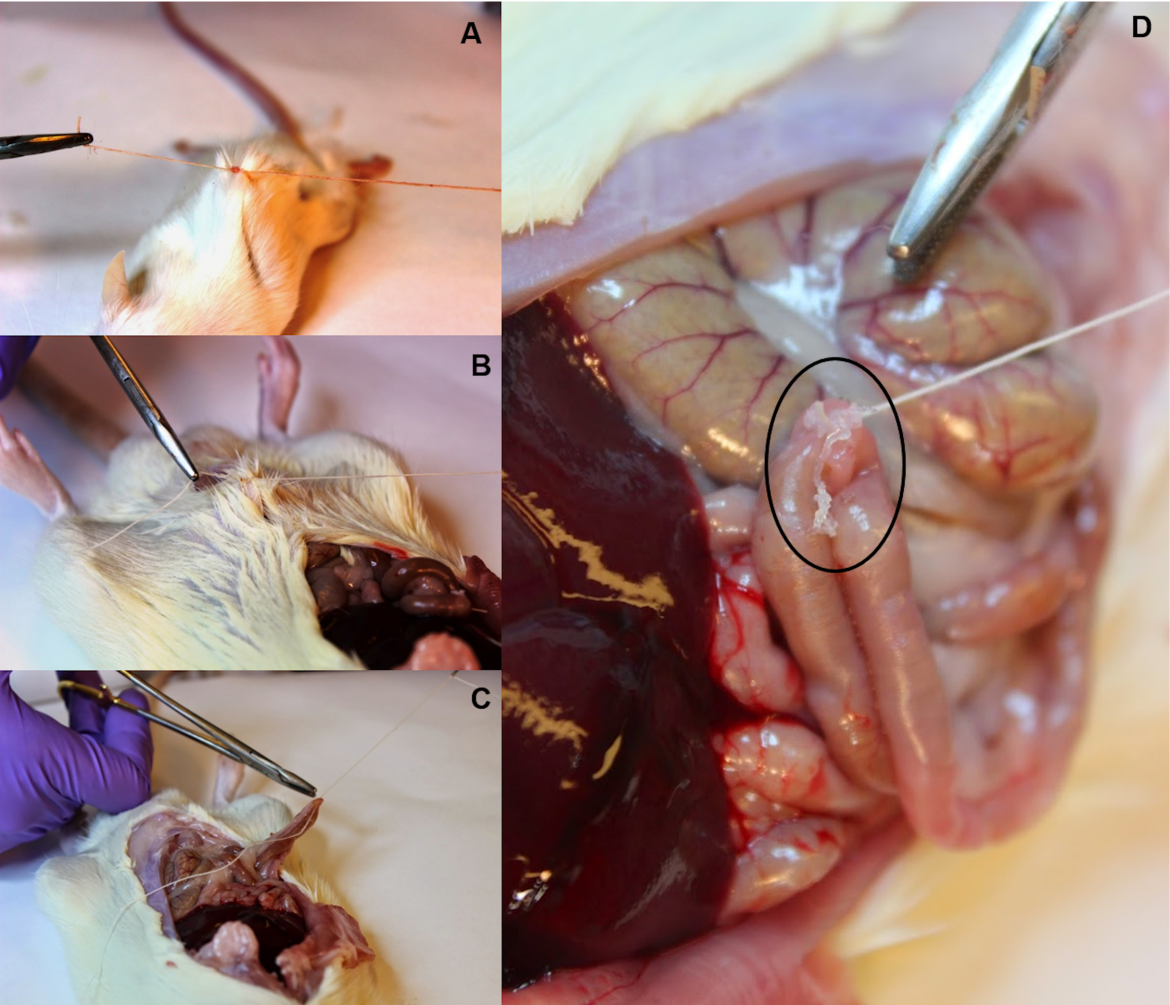
NFCA coated suture performance testing on small-animal tissues. Suturing was completed with instrument type knots. 12 out of 14 successful sutures were performed on mouse and rat skin (A and B) in addition to rat intestine (C). Some peeling off was observed in 2 sutures during the performance testing (D). Failed sutures were clearly visible and easily noticed as long tube-like segments (D).

NFCA coating of standard absorbable surgical sutures were performed as described above. Sutures were cross-linked with calcium chloride and barium chloride before attempting to suture various internal organs and tissues of freshly sacrificed mice and a rat. The fabricated threads felt slightly more rigid than a standard thread when performing the suturing operation. Mouse and rat soft tissues: spleen, liver and muscle tissue S2 Fig, in addition to intestine and skin (Fig 6) were sutured (n = 14). Instrument knot tying was performed to complete the sutures on all tissue samples.

Two sutures from a total of 14 attempts showed slight peeling off in the NFCA coating. Failed sutures were noticed during the attempts, as the detached coating segments were apparent to the naked eye. However, 12 attempts were successful and knot tying was performed on sample tissues without any peeling off (Fig 6). Peeling off was observed on one rat intestine and one skin suture sample and none on mice.

## Discussion

The NFCA threads were relatively easy to handle after crosslinking and could be bent and made into knots without breaking apart, as our group has previously reported that glutaraldehyde crosslinking made NFC threads slightly more brittle [32]. Hydrogel was injectable at 7–9% alginate concentrations. The addition of alginate showed an increase in both loss and storage moduli strengthening the hydrogel network roughly by one order of magnitude. The ratio of the moduli remained similar to the native NFC hydrogel, suggesting that the NFCA mixture retained its elastic properties. Mechanically the native NFC hydrogel resembles a collagen network, thereby providing good conditions for cell culture [9,15]. Rheological measurements showed that the effect on increasing the alginate content from 8% to 9% was minor in terms of gel strength and viscosity. However, the addition of alginate did improve the gel composition and enabled crosslinking. Viscosity values were 77%, 83% and 87% higher with 7%, 8% and 9% NFCA’s respectively than with NFC alone. A similar increase was observed with storage and loss moduli values. For example, the 8% NFCA shows an increase in storage modulus from low to high frequencies at 87% and 100% respectively. Alginate content lower than 7% would probably result in a weaker hydrogel composition and therefore be unfit for suture coating fabrication. Additionally, alginate stabilized the hydrogel structure which can be seen from the frequency sweep measurements as the NFCA hydrogels were relatively independent of the angular frequency at investigated ranges as opposed to NFC alone at higher frequencies, where some curve shifting could be observed. Further addition of alginate (over 9%) made injectability problematic and the final amount of 8% (w/v) alginate content was found to be optimal in terms of strength and the thread fabrication process itself. Therefore, according to the rheological results and injectability, the coating composition of 1.35% NFCA and 8% alginate content was used with the cell culture studies and the suture performance tests.

HepG2 cells maintained almost 100% viability as shown in the confocal images. Cell morphology showed limited hepatic cluster like growth when entrapped within the thread matrix, probably due to the restricted space within the dense NFCA network. However, the cells were stable and viable for up to 2 weeks of culturing, indicating a proper flow of nutrients and gas exchange. Typical hepatic spheroid formation within the thread would probably require a more dilute NFC mixture, as shown previously [10,43]. However, the lower viscosity should be addressed to compensate the loss of strength from the increasing amount of water content. HepG2 cells seeded on the surface of the thread showed a distinguished hepatic morphology and growth in clusters that increased in size during the 2-week study period. For surface culture, collagen treatment was necessary. Without collagen, the cells did not adhere properly on the surface of NFCA. Therefore, modifications are required for surface cell attachment. To avoid the use of collagen, this could be solved by, for example, including cell-adhesion peptides in the NFC matrix [44].

Model co-cultures performed with dual HepG2 and SK-HEP-1/HepG2 cultures expressed their typical respective morphologies correctly depending on the cell type. SK-HEP-1/HepG2 co-cultures were examined after the 48-hour incubation period and the formation of a monolayer was easily distinguished on the surface of the NFCA threads. However, due to the restricted incubation period, which was limited by the imaging timeframe of the cellular dye, SK-HEP-1 monolayer was not fully confluent. Longer incubation periods or NFCA modifications could improve cell attachment to achieve better confluence. For example, an effective bioconjugation method has been shown to improve cell-adhesion of nanofibrillar cellulose surfaces [45]. Therefore, NFCA threads have potential as cell-carrier systems for cell-based therapy, such as wound-healing platforms, where cell migration could be mediated from the suture to integrate the post-surgical treatment into the surgical process. Cell delivery can be facilitated from within the thread, forming a protective layer to improve cell viability and decrease unwanted cell distribution, or alternatively from the thread surface for more immediate cellular interactions. Additionally, with this finding we have shown that the NFCA threads enable multiple types of cells to be delivered simultaneously to the target site.

Coating surgical sutures with NFCA was shown to be a simple and reproducible process. The total hydrogel coated suture thickness was measured at 297±25 μm (n = 10), which corresponds closely with the USP designated diameter for a standard 2–0 synthetic absorbable suture (300 μm). Variation in diameter of all coated sutures was 8.2% of total diameter, which was small enough to enable suturing successfully with 12 out of 14 attempts. Average weight of the threads before and after coating were 0.86±0.04 mg/cm and 1.96±0.27 mg/cm respectively, which results in a small total increase in weight of 1.1 mg/cm in comparison of uncoated and coated sutures. As an example, a full 36-inch single coated suture would only weigh approximately 100.1 mg more than a suture without the coating. Therefore, the total weight increase was not very significant. HepG2 cells were shown viable within the coating material during the 72-hour incubation period as expected from the previous 2-week NFCA thread viability results. It was noticed that the polyester suture had affinity to the dye which was shown clearly after the coating was removed from the suture. Suture coating could be peeled off as large intact tube like segments while entrapping the cells within. This suggests that the tube remains at the target site after suture degradation, prolonging the cellular activity. The fabrication method enables the entire coating of a suture; however, the small variation in thickness may cause the coating to peel off in addition to choosing the correct needle. 2–0 compatible needles should be used to compensate the coated 3–0 diameter.

Small-animal suture performance tests showed peeling off on two samples (rat skin and intestine) from a total of 14 attempts. Variations in thickness during the coating process or incorrect needle size could cause the coating to get stuck in the tissue at the beginning of the suturing operation; especially with more penetration resistant tissues, such as the skin. The synthetic poly lactic-co-glycolic acid (PLGA) co-polymer sutures used in this study have been reported to have hydrophobic properties [46]. Hydrophobic sutures could present problems with the high water content of the NFCA coating. The hydrophobicity of the synthetic PLGA sutures could explain the observed peeling off effect as large intact tube like segments. Other types of sutures (i.e. less hydrophobic) should improve the performance of the coating material. However, 12 sutures were performed successfully with various tissues including instrument type knots to close the sutures. Furthermore, the NFCA thread coatings were flexible and strong enough to enable and withstand the mechanical stress of the suturing process itself without breaking apart, from the beginning of the operation to the closing of the knot at the end of each suture.

The NFCA coated sutures have potential to combine treatment with surgical suturing where cell therapy is viable, removing the need for additional injections of cells to target sites or into the sutures themselves, which can result in suboptimal viability and uncontrolled cell distribution, especially with unprotected cell seeded sutures [4]. The number of cells could be controlled by the concentration of cells within the coating in thread thickness and length, in addition to protection and enhanced target cell delivery. The optimal number of cells in cellular delivery depends on its application; our experiments were done with 1.5 * 10^4^ cells/cm/suture. In comparison to sutures seeded with cells, a study for cardiac therapy after infarcted myocardium contained 5.9 * 10^3^ cells/cm/suture [3], and in a study for post-operative complications after tracheal surgery contained 4.0 * 10^4^ cells/cm and 1.3 * 10^4^ cells/cm [47].

However, while the native NFC is non-toxic and biocompatible, the biodegradability in the body is a concern; therefore, additional modifications could be made into the NFC structure to enable self-degradation. Alternatively, embedding the surgical suture, or the NFCA matrix itself, with cellulases, which have proven to be effective in degrading NFC and have not shown cytotoxic effects in stem cells [10]. In addition, commonly used polymers in sutures provide slightly acidic conditions as they degrade into lactic or glycolic acids [48], which would improve cellulase enzyme performance as their relative activity increases with lower pH [49]. However, the absorbable suture is biodegradable and Velosorb, according to the manufacturer, loses its total tensile strength within 2 weeks post-surgery, a time period which the cells can easily survive according to our findings in this study. Additionally, the cross-linked alginate will dissolve with the release of divalent Ca^2+^ and Ba^2+^ ions [23], therefore, eventually loosening the system and allowing the cells to distribute and interact with the surrounding tissue directly.

According to our findings, we propose that NFCA coated surgical sutures are viable on easily accessible areas, such as the skin and other surfaces where the removal of NFC would not cause further complications, i.e. the gastrointestinal tract, where the rapid renewal rate and motility would eventually remove traces of NFC. NFCA coated sutures show potential as cell-carrier systems integrated with surgical suture processes; however small-animal tissue composition and structure varies from human tissue and requires further investigation.

## Conclusions

We have demonstrated a method of producing NFCA threads and surgical suture coatings including single type cell cultures and co-cultures simultaneously within and on the surface of NFCA that are viable for up to 2 weeks. Cells on the surface show typical morphologies of their respective types (i.e. cluster and monolayer); cells inside the thread were observed as smaller clusters. Rheological measurements of NFCA showed similar viscoelastic properties when compared to the native NFC, which has been shown to support good cell culture conditions as the structural properties resemble the physiological conditions of a soft tissue collagen network. Hydrogel strength was improved with the addition of alginate to form proper suture coatings. 12 out of 14 surgical sutures coated with NFCA performed well with various mouse and rat tissue. In 2 sutures, peeling off of NFCA was observed. With cell-carrier suture systems, additional injections could be avoided while still introducing the cells into the diseased tissue. NFCA coating acts as a biomaterial for cellular growth and protection, while controlling the number of cells within. Multiple types of surgical sutures could be used for cell entrapment as coating applications. We believe that cell-carrier systems, such as the NFCA suture coatings, would address some of the issues in therapeutic cell delivery, such as reducing undesired cell migration, aggregation and low survivability. In addition, the coating method allows co-cultures within the same suture, which could prove beneficial in future cellular therapies.

## Supporting information

S1 Fig HepG2 cells seeded on top of NFCA hydrogels and stained with live (green) and red (dead). A) Confocal imaging after 1-week incubation. B) 1-week incubation, HepG2 cells killed with 70% ethanol. C,D) Confocal images after 2-week incubation period showing large hepatic clusters.
(TIF)

S2 Fig NFCA coated suture performance on freshly sacrificed mice. Mouse liver (top), spleen (middle) and muscle tissue (bottom) were successfully sutured without NFCA coating peeling off.
(TIF)
