# Supplementary figures and images for: Nanofibrillar cellulose-alginate hydrogel coated surgical sutures as cell-carrier systems
Source: PLoS One. 2017 Aug 22;12(8):e0183487. doi: 10.1371/journal.pone.0183487 (PMC5567492; doi:10.1371/journal.pone.0183487)

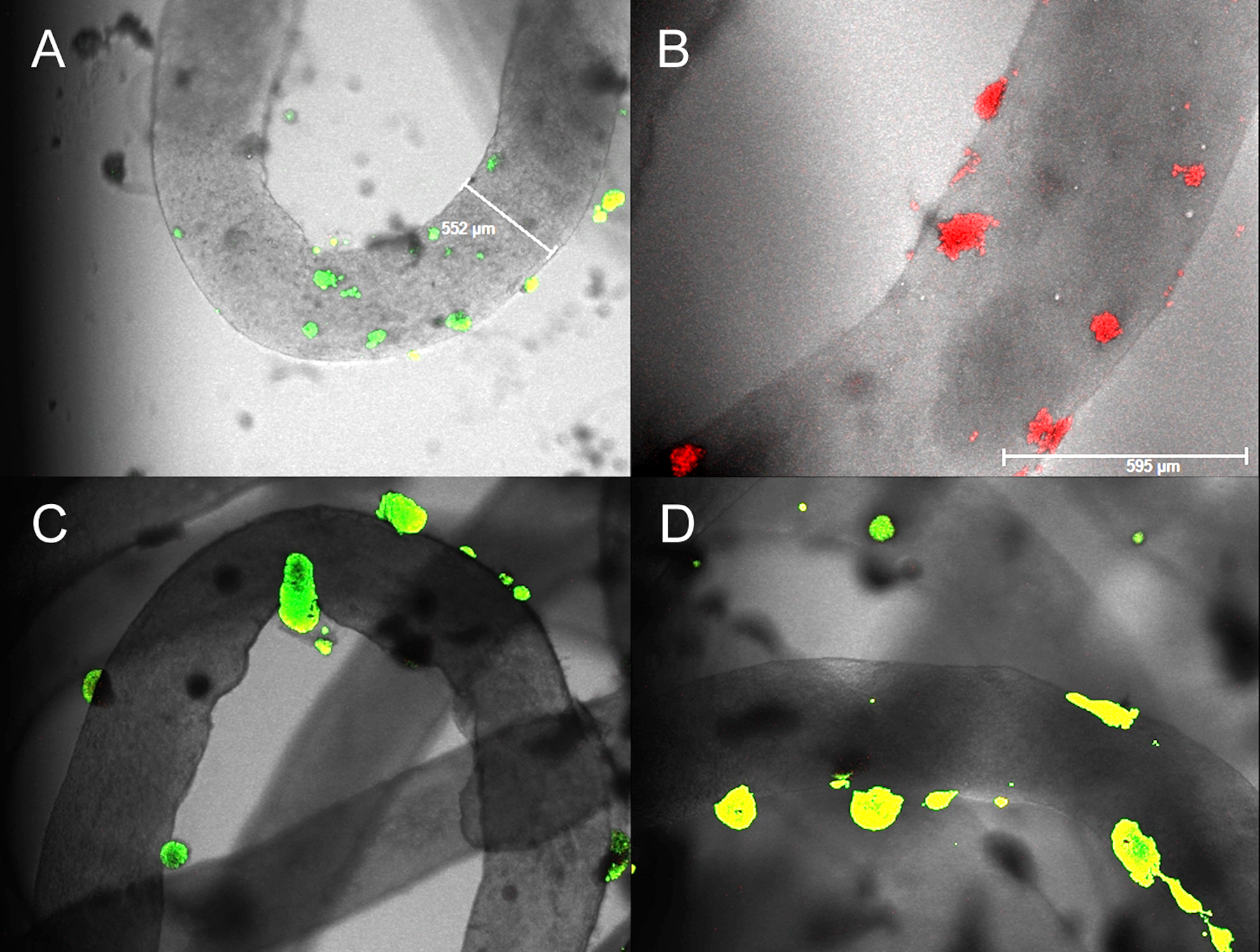

Supplement: S1 Fig — A) Confocal imaging after 1-week incubation. B) 1-week incubation, HepG2 cells killed with 70% ethanol. C,D) Confocal images after 2-week incubation period showing large hepatic clusters. (TIF) [file pone.0183487.s001.tif]

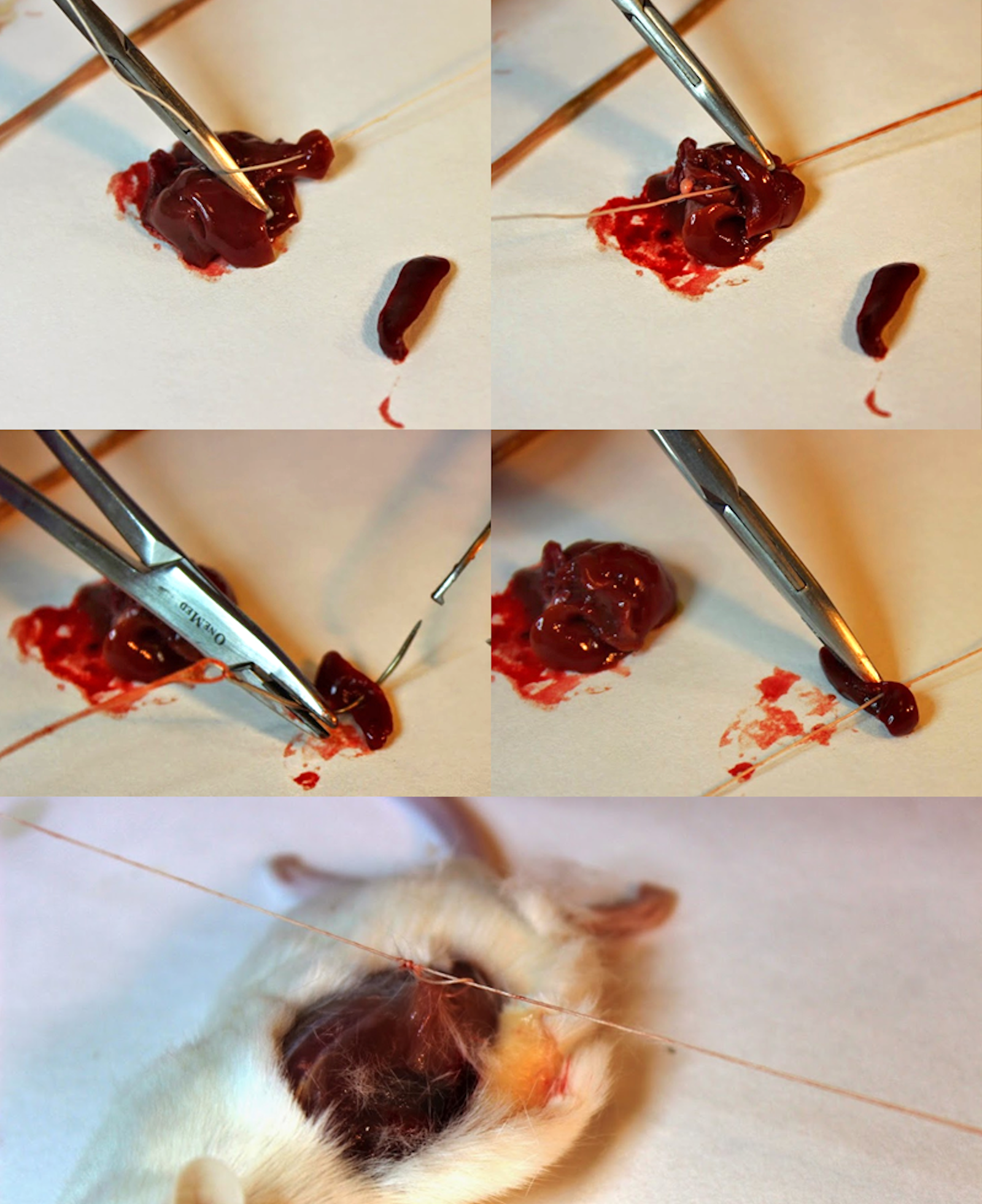

Supplement: S2 Fig — Mouse liver (top), spleen (middle) and muscle tissue (bottom) were successfully sutured without NFCA coating peeling off. (TIF) [file pone.0183487.s002.tif]
